# Correlation of Symptoms of Anxiety-Depressive and Obsessive-Compulsive Spectrum and BMI in Patients with Eating Disorders

**DOI:** 10.1192/j.eurpsy.2025.1385

**Published:** 2025-08-26

**Authors:** M. S. Artemieva, A. M. Vasilyev, D. A. Yudelevich

**Affiliations:** 1 Psychiatry and Medical Psychology, RUDN University, Moscow, Russian Federation

## Abstract

**Introduction:**

Eating disorders (EDs) are characterized by intrusive thoughts about food, weight loss, and body image, often accompanied by compulsive behaviors related to weight control. Obsessive-compulsive behavior is frequently observed in individuals with EDs. The relationship between obsessive-compulsive disorder (OCD) and EDs is complex due to overlapping symptoms. Research indicates that individuals with both OCD and EDs are prone to depression and anxiety, which may manifest as secondary responses to stress. Symptoms of OCD can significantly impair functioning, leading to maladjustment. Some studies have noted a correlation between increased body mass index (BMI) and reduced depressive symptoms in patients suffering from anorexia nervosa.

**Objectives:**

This research aimed to investigate the severity of anxiety-depressive and obsessive-compulsive symptoms in relation to changes in BMI among patients with eating disorders.

**Methods:**

The study was conducted at the Center for Eating Disorder Research in collaboration with the Department of Psychiatry and Medical Psychology at the Peoples’ Friendship University of Russia. A sample was created from patients undergoing inpatient treatment with diagnoses according to ICD-10 (F50.0, F50.1). Clinical interviews were conducted during the first and fourth weeks using PHQ-9 (for depression), GAD-7 (for anxiety), and the Yale-Brown Obsessive Compulsive Scale. Statistical analysis was performed using Jamovi 2.3.28.

**Results:**

Thirty female patients participated in the study. All received psychopharmacotherapy. The average age was M=14.4, SD=1.5. The mean BMI at the start was M=13.3, SD=1.69, increasing by M=1.20, SD=0.612 by the second assessment. Depressive symptoms were observed in 28 (93.33%) during week one and in 25 (83.33%) during week four. Elevated anxiety levels were noted in 24 (79.99%) during week one and in 25 (83.33%) during week four. Significant OCD symptoms were present in 25 (83.33%) during week one and in 21 (70%) during week four. Correlation analysis revealed no significant relationships (p > 0.01) between BMI levels and OCD symptoms on the Yale-Brown scale (r = 0.099), depressive symptoms on the PHQ-9 scale (r = 0.28), or anxiety levels on the GAD-7 scale (r = 0.369).

**Conclusions:**

Findings indicate certain relationships between BMI and psycho-emotional states among patients with eating disorders; however, statistically significant correlations were not identified (p > 0.01). This underscores the need for further research to deepen understanding of these relationships, especially since none of the respondents achieved normal BMI values (18.5). Future studies should involve larger sample sizes and extended time frames for more reliable data.

**Disclosure of Interest:**

None Declared

